# Spatiotemporal optical vortex reconnections of loop vortices

**DOI:** 10.1515/nanoph-2024-0594

**Published:** 2025-02-04

**Authors:** Jordan Adams, Andy Chong

**Affiliations:** RF and Optics, Riverside Research Institute, Beavercreek, OH 45431, USA; Department of Physics, 34996 Pusan National University, Busan, 46241, Republic of Korea; Institute for Future Earth, Pusan National University, Busan, 46241, Republic of Korea

**Keywords:** spatiotemporal vortices, loop vortices, vortex reconnection

## Abstract

Reconnections of spatiotemporal optical vortices have been shown to occur between line vortices. Here, we show that reconnections also occur between spatiotemporal loop vortices in optical waves. As optical loop vortices propagate in a media with spatial diffraction and material group velocity dispersion, unique reconnections occur. The birth and death of loops can occur, with certain loop vortices emerging from or collapsing to a single point while interacting with others. As certain parameters are varied in the model, complex arrangements of loops form in space-time from simple initial fields.

## 1 Introduction

Vortex reconnections are observed in a variety of systems such as viscous fluids [1], [2], [3], [4], [5], superfluids [6], [7], liquid crystals [8], Bose–Einstein condensates [9], superconductors [10], [11], and magnetohydrodynamics [12]. Two vortices approach each other and as they intersect, they split and reconnect with the other vortex. Reconnections can give rise to some physical phenomena. For example, reconnections can cause turbulence in viscous fluids that leads to aeroacoustic noise [1] as well as other interactions in superfluids [6], [7]. Magnetic reconnections occurring in plasma accelerate charged particles and are behind magnetospheric substorms and solar flares [12].

Vortex reconnections also occur in optical waves. Three-dimensional arrangements of vortices can occur in the monochromatic light propagation path, but reconnections can only be seen in time-varying media or by varying input parameters [13], [14]. In contrast, we recently demonstrated that spatial and spatiotemporal line vortices in polychromatic wavepackets can undergo reconnections dynamically during propagation [15]. Spatiotemporal vortices are a new type of optical vortex, with angular momentum transverse to the propagation direction. A review and tutorial on spatiotemporal vortices can be found in ref. [16].

In addition to line vortex reconnections, reconnections with closed loop vortices have been demonstrated in many areas of physics [3], [7]. In optics, loop vortex reconnections have been observed in monochromatic waves with time-varying randomness in the field [14]. In such a case, loop vortex birth or death occurs where loops can grow from or collapse to a single point. In addition to this, spatiotemporal loop reconnections were recently observed in nonlinear pulse propagation in plasmas [16]. However, it is unknown how spatiotemporal loop reconnections unfold in linear free-space propagation, nor is a pathway known for generating such reconnections. Therefore, developing a linear model for studying loop reconnections could give valuable insight into a variety of fields of physics.

Here, we present spatiotemporal vortex loop arrays that undergo reconnections in polychromatic optical wavepackets with linear propagation. While many possible loop arrangements could undergo reconnections, here we will concentrate on the most relevant cases that share similarities to those observed in nonlinear optics and fluids. Considering spatiotemporal ring vortices in the Fourier domain provides a convenient approach for studying the loop vortex reconnections. The Fourier transform of a single ring vortex is a paraboloid amplitude null with a *π* phase shift. By combining such paraboloids in the Fourier domain, we can obtain the interactions of multiple loop vortices in space-time that undergo reconnections with propagation. The two-paraboloid reconnection case is similar to experimental results of spatiotemporal loop reconnections found in nonlinear pulse propagation [17] while the three-paraboloid case resembles the multiring collisions in viscous fluids [3]. Additionally, the birth and death of loop vortices are also observed in dynamic reconnection processes.

As loop vortex reconnections can be studied from a simple linear propagation model, we believe this work could give insight into loop vortex reconnection governed by nonlinear equations in fluids or other media. With such important phenomena like turbulence or space weather being driven by reconnections, the understanding gained from reconnections in optics could have a large impact. Additionally, due to the complex arrangements of vortices that can be obtained from simple patterns in the frequency domain, we believe future research into applying these loop reconnections as information carriers for optical free-space communication is warranted. As these loops are highly coupled in space and time, the profiles could have unique benefits for propagation through turbulence and future work should explore the resilience of these loops to propagation through random media. Finally, as the suggested profiles are relatively simple in the frequency domain, these reconnections and loop arrangements can be generated using recently demonstrated experimental setups for 2D+1 amplitude and phase control [18].

## 2 Results

### 2.1 One paraboloid amplitude null

To study vortex reconnection phenomena, we begin with the field in the space-time domain, which is given by the Fresnel transform of the particular function in the spatial frequency–frequency $\left(k_{x},k_{y},\omega \right)$ domain as shown in Equation (1). In this manuscript, we will refer the $\left(k_{x},k_{y},\omega \right)$ domain as the Fourier domain.

(1) $$\begin{array}{ll} E\left(x,y,t\right) & =F_{3D}\left\{{\left(k_{x}^{2}+k_{y}^{2}+d\omega \right)}^{l_{1}}{\left(k_{x}^{2}+d^{2}\omega^{2}+k_{y}\right)}^{l_{2}}\right.\\ & \;\times \left.{\left(k_{y}^{2}+d^{2}\omega^{2}+k_{x}\right)}^{l_{3}}\right.\\ & \;\times \left.\exp\left(-i\pi \left(A_{k_{x}}k_{x}^{2}+A_{k_{y}}k_{y}^{2}+A_{\omega }\omega^{2}\right)\right)u\right\} \end{array}$$

where ℱ_3*D*_ represents the 3D Fourier transform, $u=\exp\left(-\frac{k_{x}^{2}}{w_{k_{x}}^{2}}-\frac{k_{y}^{2}}{w_{k_{y}}^{2}}-\frac{\omega^{2}}{\Delta \omega^{2}}\right)$ is the spatial and temporal frequency envelope, *w* is the spatial frequency bandwidth, Δ*ω* is the temporal frequency bandwidth, *d* = *w*/Δ*ω* is a factor that compensates the frequency/spatial frequency bandwidth mismatch, and the $l=\left\{l_{1},l_{2},l_{3}\right\}$ are the paraboloid powers. Additionally, the coefficients $A_{k_{x}},A_{k_{y}},A_{\omega }$ control diffraction and dispersion effects for various propagations conditions. For example, focusing along *x* by a cylindrical lens would have $A_{k_{x}}=\lambda z$ while *A*_*ω*_ would be constant and $A_{k_{y}}$ approximately constant.

It is worth noting the meaning of the first three terms. For instance, when the first term is set to zero $\left(k_{x}^{2}+k_{y}^{2}+d\omega =0\right)$ , it defines a paraboloid. On the paraboloid surface, the electric field becomes zero and, therefore, the amplitude will be null. At the same time, there is a sign change from the inside of the paraboloid to the outside, indicating the *π* phase shift on the paraboloid surface for odd powers of *l*. Consequently, the first three terms represent three perpendicular paraboloids with amplitude null and a *π* phase shift in the Fourier domain.

From this simple Fourier domain representation, complex reconnections occur in space-time with propagation under various combinations of the paraboloid powers, spatial diffraction effects, and temporal dispersion effects. For the simplest case of $l\;=\;\left\{1,0,0\right\}$ , there is a single paraboloid that gives one ring vortex in space-time. For simplicity, we assume a circular symmetric beam with bandwidth $w=w_{k_{x}}=w_{k_{y}}$ and frequency bandwidth $\Delta \omega \;=\;\frac{w}{d}\;=\;w$ (e.g., d = 1 mm^−1^/1 THz), which results in equation (1) at the focus $\left(A=0\right)$ becoming

(2) $$\begin{array}{ll} E\left(x,y,t\right) & =\left(x^{2}+y^{2}-\frac{1}{\pi^{2}w^{2}}+i\frac{t}{w\pi }\right)\pi^{5/2}gw^{7}\\ & \;\times \exp\left(-\frac{1}{w^{2}}\left(x^{2}+y^{2}+c^{2}t^{2}\right)\right) \end{array}$$

**Figure 1:**
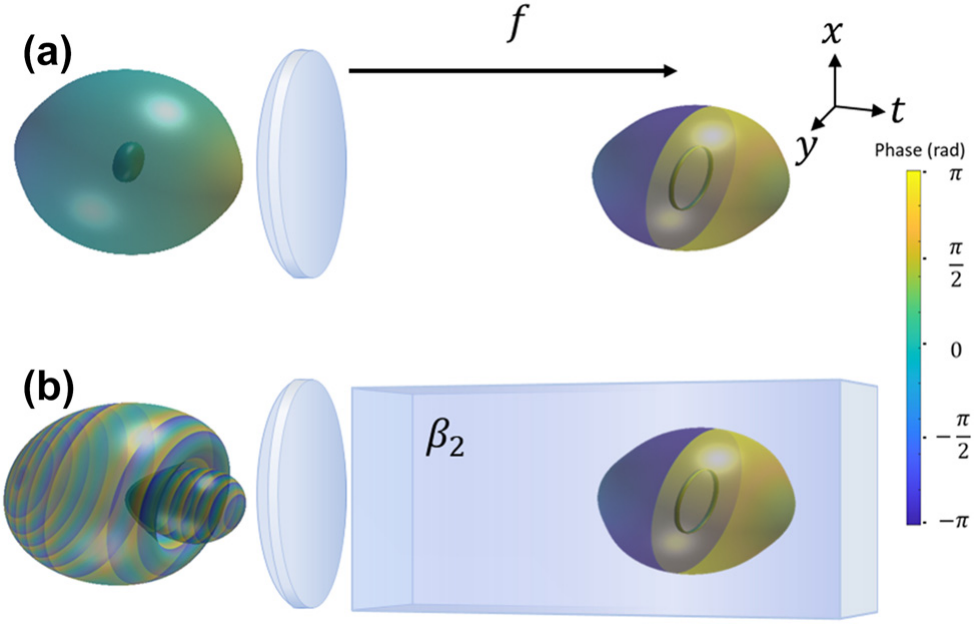
Illustrative examples of generating single loop vortices, highlighting an input wavepacket’s relationship to the resulting loop’s frequency domain profile. (a) A ring vortex forming at a spatial focus arises from an intensity null centered around a point. (b) A ring vortex forming at the spatial and temporal focus starts from a highly chirped paraboloid amplitude null.

This is a single closed-loop vortex with size dependent on the bandwidth. As an example, if this loop vortex was created at the focus of a focusing lens, the input before the lens would be $E_{in}\propto \left(x^{2}\;+\;y^{2}\;+\;it\right)u^{\prime }$ , which has phase $\Gamma =atan\left(\frac{x^{2}\;+\;y^{2}}{t}\right)$ (Figure 1a). This has an intensity null centered around a point and is roughly ellipsoidal in shape. Additionally, the phase only advances with varying the polar coordinate $\theta \;=\text{ atan}\left(\sqrt{x^{2}+y^{2}}/t\right)$ , which reveals the loop with poloidal increasing phase evolves to a point with spherical-like phase profile. As another example, if the optical wavepacket is temporally focused during propagation in dispersive media, with the temporal focus effect coinciding with the spatial focus effect, the input wavepacket would be highly chirped with a paraboloid amplitude null (Figure 1b).

### 2.2 Two perpendicular paraboloid amplitude nulls

With two paraboloid amplitude nulls in the Fourier domain $\left(l\;=\;\left\{l_{1},l_{2},0\right\}\right)$ , multi-loop arrays are generated, leading to complex reconnections during propagation.

In the first example, we consider spherical focusing in dispersive media with simultaneous spatial and temporal focusing. For simplicity, we assumed the same propagation (focusing) effect in all directions with $A\;=\;A_{k_{x}}\;=\;A_{k_{y}}\;=\;A_{\omega }$ where *A* = 0 represents the wavepacket at the focus, and for now set the bandwidths to $w_{k_{x}}\;=\;w_{ky}\;=\;1\;\text{m}{\text{m}}^{-1}$ and Δ*ω* = 1 THz.

**Figure 2:**
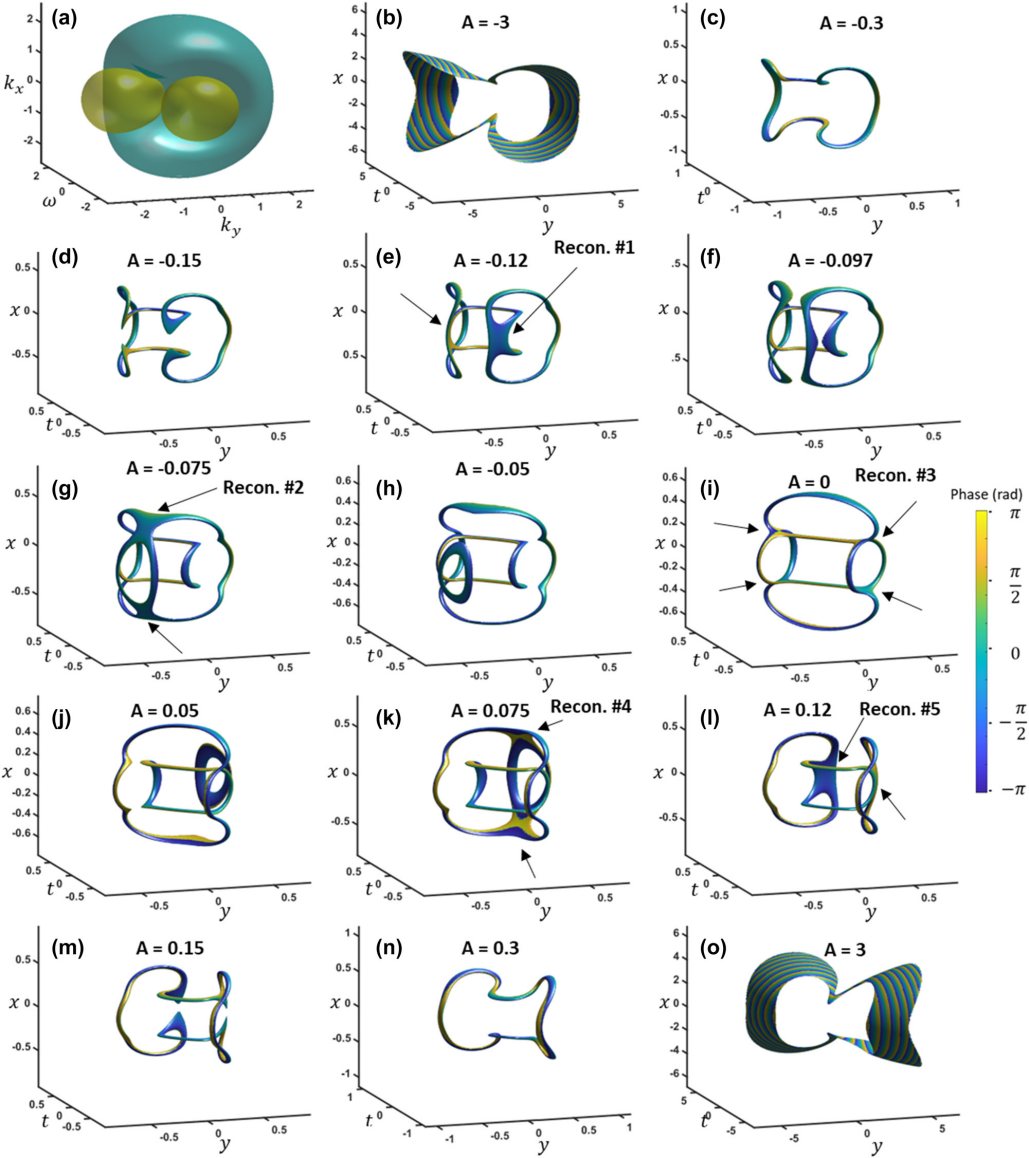
Reconnections resulting for simulteanous spatial and temporal focusing for the case of two paraboloids in the frequency domain. (a) The two paraboloids in the frequency domain. (b)–(o) Inside the wavepacket envelope for different values of *A* for spatiotemporal focusing, with (g) showing the focus. The iso-surface values are 20 % normalized intensity. Phase is shown from −*π* to *π*. *k*_*x*_ and *k*_*y*_ units are in inverse millimeters and *ω* units are in THz, while *x* and *y* units are in millimeters and *t* is in picoseconds. *A* is in units of mm^2^ and ps^2^ for the spatial and temporal components, respectively. Also shown in Video 1.

Figure 2a shows the iso-intensity surface profile with two perpendicular paraboloid amplitude nulls in the Fourier domain. Figure 2b presents the corresponding space-time iso-intensity plot at large negative *A*. We remove the wavepacket envelope for clarity by using a filter and display the resulting processed intensity shown at a 20 % iso-value. The two paraboloidal sheets merge and collapse into a closed-loop vortex as *A* increases from *A* = −3 to *A* = −0.3 (Figure 2b and c). From *A* = −0.15 to *A* = 0, a two-step reconnection sequence occurs via folding and releasing of multiple loops. The set of reconnections are labeled in the figures along with each individual reconnection being highlighted with arrows. In this process, one loop shifts away and shrinks until it disappears (Figure 2h and i). This process is referred to as vortex death [14]. Figure 2i shows that at the focus a multi-loop connection forms. As *A* increases further, the reverse process occurs, leading to the birth of a vortex loop on the opposite side of the wavepacket (Figure 2j–o). For a clearer perspective, a video of the entire process is available in Video 1. The multi-loop array at the focus resembles the results presented in self-focused nonlinear pulse propagation [16].

**Figure 3:**
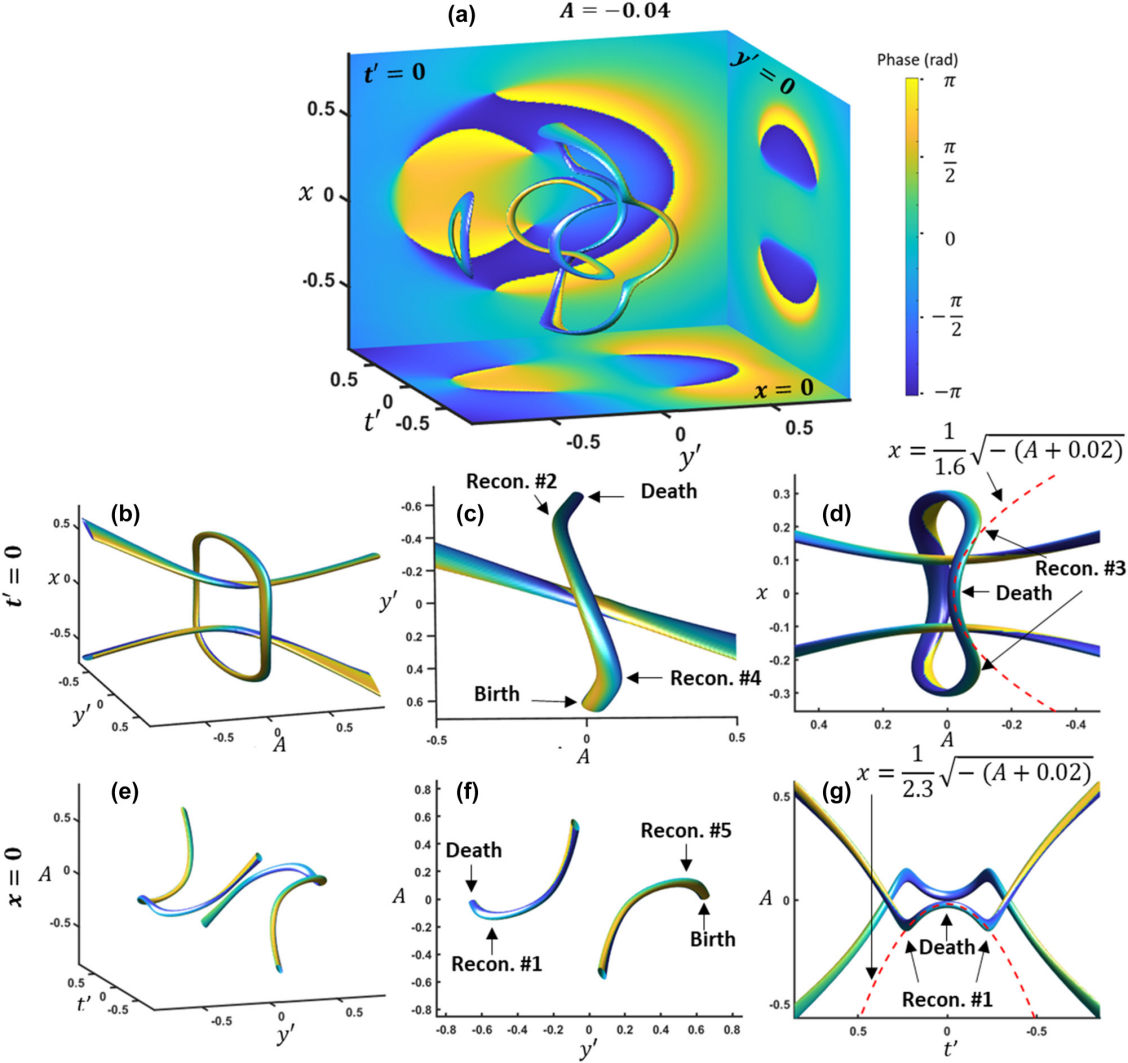
Cross-sections of loop vortices and their trajectories during reconnecting with propagation. (a) The iso-intensity at A = −0.04 in coordinates rotated by 45° in the y-t plane. Phase cross sections are displayed for *x* = 0, *t*′ = 0, and *y*′ = 0. (b)–(d) The trajectory of the top and bottom of the ring vortex is shown by plotting the iso-intensity of the *t*′ = 0 cross section over *A*. (e)–(g) The trajectory of the sides of the ring vortex is shown by plotting the iso-intensity of the *x* = 0 cross section over *A*. The iso-surface values are 20 % normalized intensity.

These ring vortices have spiral phases as shown in the cross section phase plots of Figure 3a, where the coordinates are rotated to $y^{\prime }=\frac{1}{\sqrt{2}}\left(y-t\right)$ and $t^{\prime }=\frac{1}{\sqrt{2}}\left(y\;+\;t\right)$ to obtain the best cross-sectional views. Due to the symmetry of the rings, the total orbital angular momentum (OAM) calculated by $L=\int \vec{r}\times E^{*}\nabla E\text{d}r$ with $\vec{r}\;=\;\left[x\text{,}y\text{,}t\right]$ is always zero, independent of propagation parameter *A*. Despite of the local OAM conservation, there can be motion of these vortices. For example, during a reconnection in fluids, the distance between vortices scales with time according to *d* ∝ *t* ^0.5^ [2]. It will be valuable to see if such motion occurs with spatiotemporal optical reconnections.

To analyze the trajectories, we plot the cross sections over the propagation dimension, *A*, to create a 3D trajectory. The *t*′ = 0 cross section trajectory is plotted in Figure 3b–d, which shows the trajectory of the top and bottom parts of each of the rings. With the 3D visualization of the cross section with propagation, the second and fourth sets of reconnections (shown in Figure 2g and k) are evident. One vortex from the initial reconnection shrinks until it disappears, while the other crosses the center, eventually reconnecting with another vortex that just appeared from a point. In total, this process maps out a closed loop. The trajectory approaching the vortex death or leaving the vortex birth scales to the half power, with a fit shown in Figure 3d, matching the motion of vortices in fluids. Likewise, the *x* = 0 cross section is plotted over *A* in Figure 3e and f. In this, the vortex trajectories don’t form a closed loop, but the motion approaching a vortex death or leaving a vortex birth still scales to the half power.

To understand how the spatial frequency and temporal frequency bandwidths affect the field, we look at the equation of the electric field at the spatial and temporal focus (*A* = 0), again assume *w* = *w*_*x*_ = *w*_*y*_ and Δ*ω* = *w*/*d* = 1,

(3) $$\begin{array}{ll} E\left(x,y,t,0\right) & =\left(\pi^{3/2}Bw^{7}-\pi^{7/2}Cw^{9}+\pi^{11/2}Dw^{11}\right)\;\\ & \;\times \exp\left(-1/w^{2}\left(x^{2}+y^{2}+c^{2}t^{2}\right)\right) \end{array}$$

(3.1) $$B=3-2ty\pi^{2}-i4\pi \left(t+y\right)\;$$

(3.2) $$\;C=4x^{2}+y^{2}+t^{2}-i\pi \left(x^{2}\left(y+t\right)+y^{3}+t^{3}\right)$$

(3.3) $$D=\left(x^{2}+y^{2}\right)\left(x^{2}+t^{2}\right)\;$$

**Figure 4:**
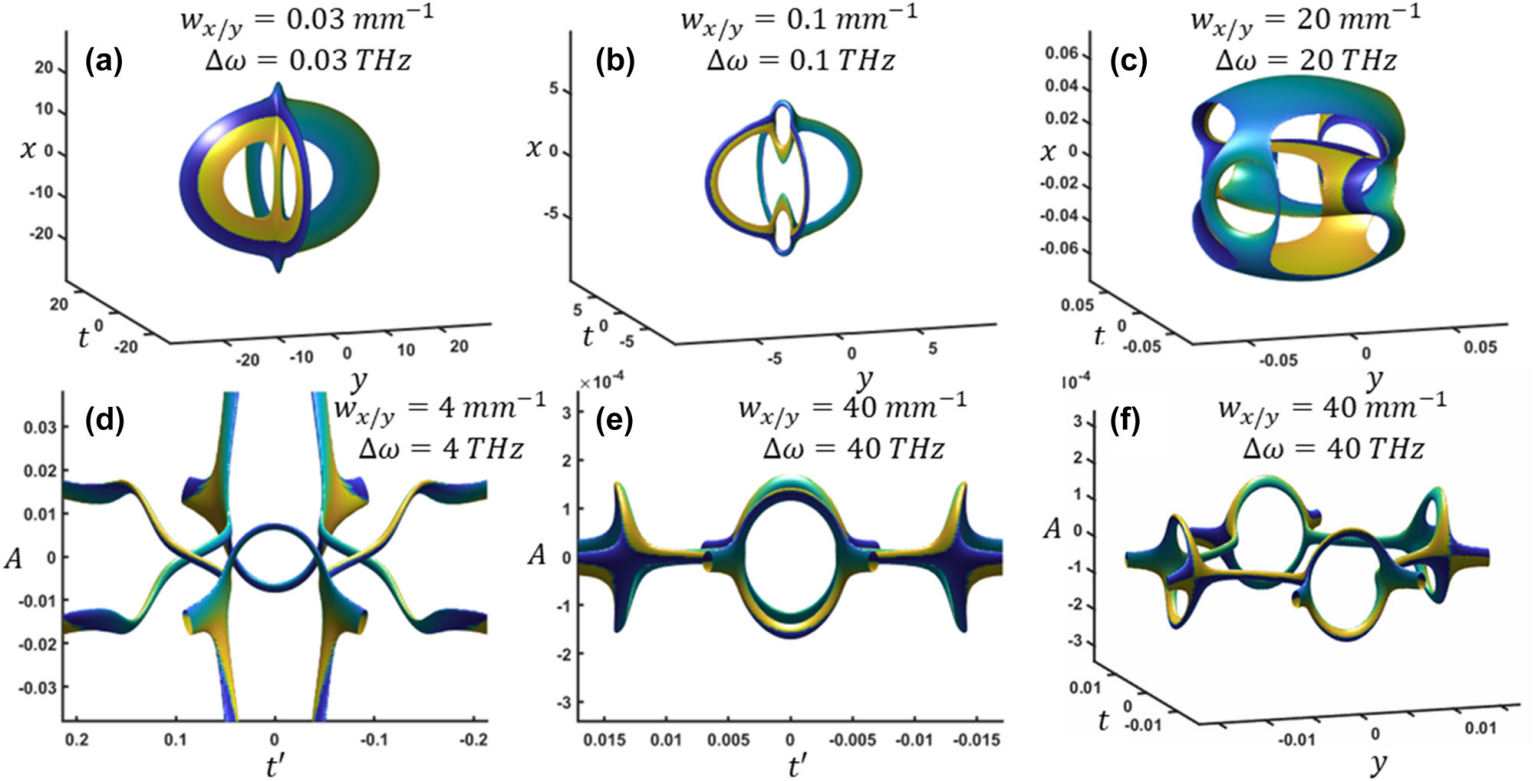
Varying the spatial and frequency bandwidth**.** (a)–(c) The iso-intensity at *A* = 0 for small to large bandwidths. (d)–(f) The *x* = 0 cross sections plotted over *A* for two different bandwidths. The iso-surface values are 20 % normalized intensity.

Equation (3) has several terms that depend on the bandwidth, *w*, which occurs because terms of different power in equation (1) (i.e., $k_{x}^{3},k_{x}^{2},k_{x}$ ) are multiplied by the bandwidth to a higher power (e.g., *w* ^3^, *w* ^2^, *w*) with a Fourier transform. This is visualized in Figure 4. At a small bandwidth, the *B* term given by equation (3.1) dominates, which is approximately a single vortex along the *x* axis (Figure 4a). As the bandwidth increases, this vortex line splits and two loops form. Further increasing the bandwidth, what was shown Figure 2i, causes the *C* term given by equation (3.2) to dominate. At large bandwidth, the *D* term given by equation (3.3) now begins to dominate equation (3), as two holes form.

It is also valuable to understand how bandwidth alters the vortex trajectory. Figure 4d–f shows the *x* = 0 cross section plotted over *A* for larger bandwidths. As the bandwidth increases, the initial reconnection is by reconnecting to other vortices (Figure 4d). With further increase in bandwidth, four symmetric rings form (Figure 4e and f). This shows that there are four simultaneous vortex births at large bandwidth, while these vortices then reconnect at eight locations at *A* = 0. Based on our analysis of varying the bandwidth in the range where term *D* dominates, the rings tend to follow the equation of an ellipse where one ring is defined as

(4) $$\frac{t^{\prime 2}}{{\left(\frac{1}{2ew}\right)}^{2}}+\frac{A^{2}}{{\left(\frac{1}{2ew^{2}}\right)}^{2}}=1$$

where *e* is the Euler number, *w* is the bandwidth, and the offset of the loop from the center is $y^{\prime }=\frac{1}{2w}$ . While locally there is a half power scaling with propagation, when there is a sequence of vortex birth, reconnection, and death, the trajectory generally can follow an elliptical path. Although the bandwidth can affect loop arrangements and trajectories, it is worthy to note that this can be compensated by changing relative coefficients on the terms in Equation (1). For example, if a coefficient *a* is added to Equation (1) so that the first two set of terms are ${\left(k_{x}^{2}+k_{y}^{2}+ad\omega \right)}^{l_{1}}{\left(k_{x}^{2}+d^{2}\omega^{2}+ak_{y}\right)}^{l_{2}}$ , the coefficient can be increased as bandwidth increases to offset this change.

**Figure 5:**
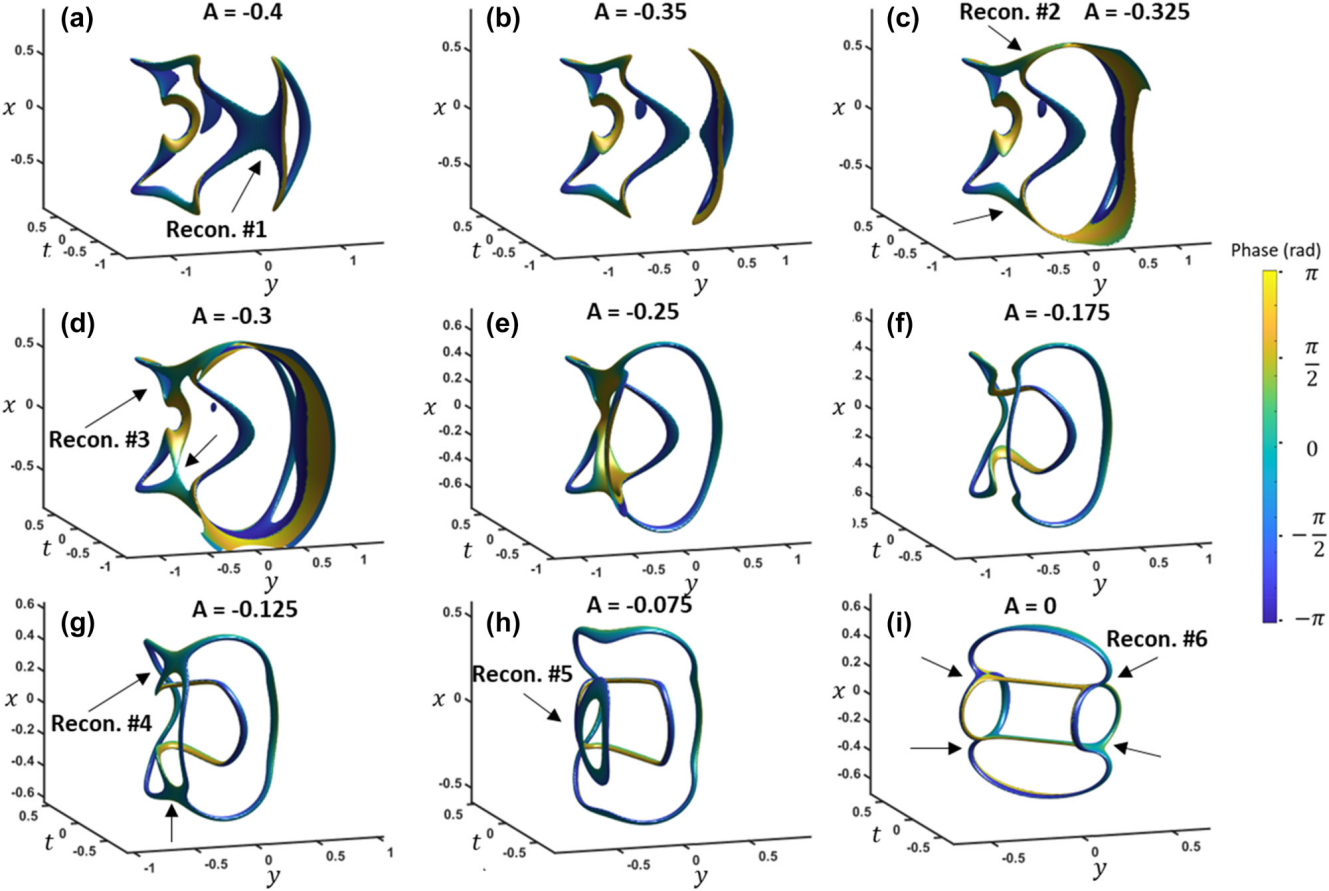
Spherical focusing to the focus for (a)–(i) increasing values of A, where (i) is at the focus. The iso-surface values are 20 % normalized intensity. x and *y* units are in millimeters while *t* is in picoseconds. *A* is in units of mm^2^. Also shown in Video 2.

Next, we show spherical focusing along the *x*-axis and *y*-axis, where we set $A\;=\;A_{k_{y}}\;=\;A_{k_{x}}$ while assuming no dispersion effect, with *A*_*ω*_ = 0, and also again using bandwidths of $w_{k_{x}}=w_{ky}=1\;\text{m}{\text{m}}^{-1}$ and Δ*ω* = 1 THz. Since the reconnections after the focus are simply the reverse process, we illustrate the propagation up to the focus (Figure 5). As the complexity of reconnections has increased significantly, we will also only present a few vortex profiles near the focus, while Video 2 captures the entire range of the process. Initially, two loop vortices reconnect and break apart into two (Figure 5a and b). They reconnect with each other and split apart again (Figure 5c–f). Near the focus, another set of reconnections occurs that leads to a similar process of vortex death (Figure 5f–i). It is also intriguing to observe how loop vortex reconnection behaves in the case of one-dimensional diffraction (i.e., cylindrical lens focusing). While we have not provided figures for such a case, we provided Video 3 and Video 4 for clearer visualization of cylindrical focusing along the *x*-axis and the *y*-axis exhibiting their unique reconnection behaviors.

**Figure 6:**
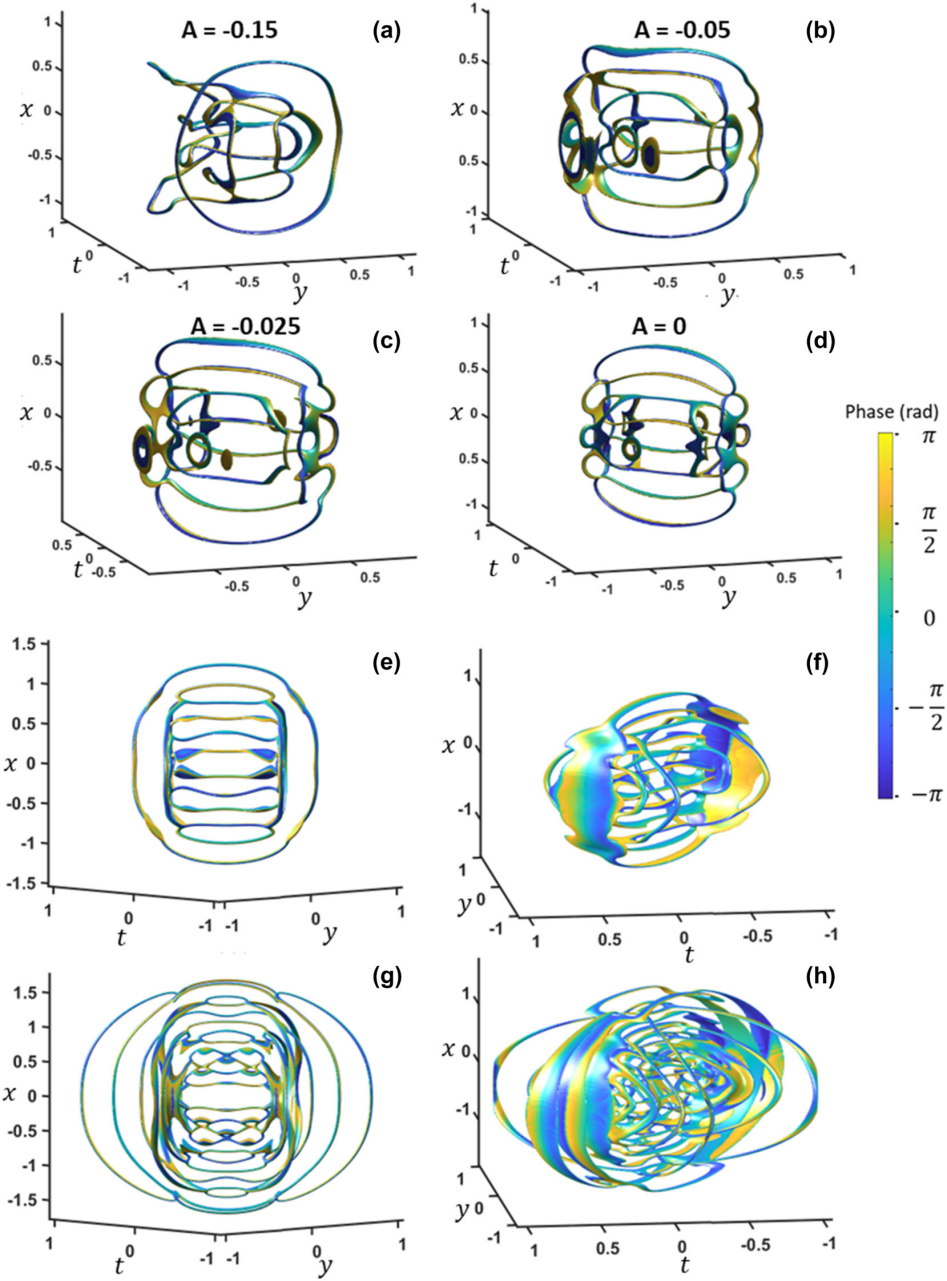
Spherical focusing with *l* = {2, 2, 0} for (a)–(d) increasing value of *A*. $\left\{l_{1},l_{2}\right\}=\left\{3,3\right\}$ from a (e) front and (f) side perspective. $\left\{l_{1},l_{2}\right\}=\left\{6,6\right\}$ from a (g) front and (h) side perspective. The iso-surface values are 20 % intensity for (a)–(d) and 15 % intensity of (e)–(h). *A* is in units of mm^2^. *x* and *y* units are in millimeters while t is in picoseconds. Also shown in Video 5.

We now examine the effect of increasing the *l* powers on the Fourier-space paraboloids. Figure 6 shows the result of spherical focusing with *l* = {2, 2, 0}, and the process is also shown in Video 5. Although much more complicated patterns emerge, at the focus, a well-organized collection of intersecting loops appears (Figure 6d). Cylindrical focusing along the *y*-axis is shown in Video 6, while spherical focusing with *l* = {2, 1, 0} is shown in Video 7. All cases exhibit distinct loop vortex reconnections.

We have also investigated increasing the powers on the paraboloids even further. Figure 6e and f shows front and side perspectives of *l* = {3, 3, 0} loop vortex reconnections at the focus. The case of powers of *l* = {6, 6, 0} at the focus is presented in Figure 6g and h. A ladder-like pattern of loops appears aligned with the *x*-axis, accompanied by two ladder patterns appear along ±45° between the y- and t-axes. We find it quite intriguing to observe these complex arrangements of loop vortices emerging from simple paraboloid amplitude null patterns.

### 2.3 Three perpendicular paraboloid amplitude nulls

Next, we investigate reconnections when three perpendicular paraboloid amplitude nulls are present in the Fourier domain using Equation (1) with *l* = {1, 1, 1}. Unique vortex reconnections occur when both spatial and temporal focusing occur simultaneously with $A=A_{k_{x}}=A_{ky}=A_{\omega }$ .

**Figure 7:**
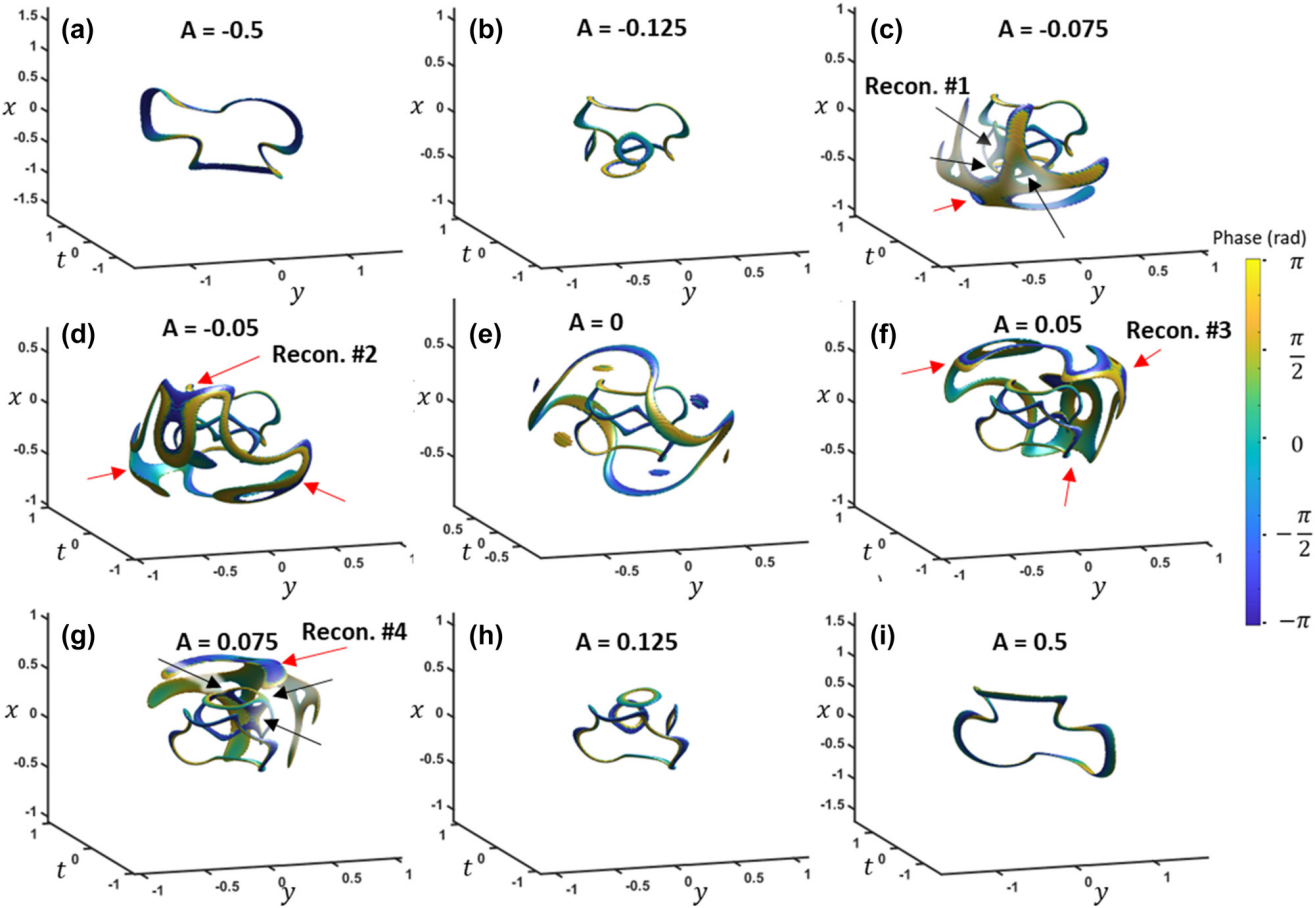
Spatiotemporal focusing of *l* = {1, 1, 1} shown from a side view (a)–(i), with (e) being at the focus. The iso-surface values are 20 % normalized intensity. *k*_*x*_ and *k*_*y*_ units are in inverse millimeters and *ω* units are in THz, while *x* and *y* units are in millimeters and *t* is in picoseconds. *A* is in units of mm^2^ and ps^2^ for the spatial and temporal components, respectively. See also in Video 8 and 9.

Figure 7a shows the iso-intensity plot with large negative *A*, where the three paraboloids have collapsed into a single closed loop vortex, similar to the one paraboloid case (Figure 2). As *A* increases, the first loop shrinks, and three loops appear below the first loop (Figure 7b). The three small loops then reconnect into two loops, where one of the two remains while the other collapses to a point (Figure 7c and d). As it approaches the focus, the smaller loop grows in size to match the original loop while remaining separated (Figure 7d and e). At the same time, an outer loop also formed undergoes reconnections with itself, eventually transforming into a similar shape as the inner two loops. The outer loop reconnections are highlighted with red arrows and the inner loop reconnections highlighted with black arrows.

After the focus, the vortex reconnection evolution is simply the reverse process seen before the focus (Figure 7f–i). The entire process is presented in more detail in Video 8 for the front perspective and Video 9 for the side perspective. The wavy closed-loop vortices that form around the focus resemble the experimental results of colliding ring vortices in viscous fluids [3]. In that case, several fluid ring vortices were propagated toward each other. After the collision, reconnected wavy ring vortices formed and quickly dissipated into turbulence. In contrast, our case of linear free-space propagation of an optical wavepacket allows reconnected loops to remain stable. This, in turn, can provide clearer visualization of the dynamics of reconnections.

Just like in the two-paraboloid case, spherical or cylindrical focusing results in more complex sequences of reconnections. The spherical focusing case can be observed in Video 10 and Video 11 from different perspectives. The cylindrical focusing along the *y*-axis also results in additional unique reconnections (Video 12 and 13).

**Figure 8:**
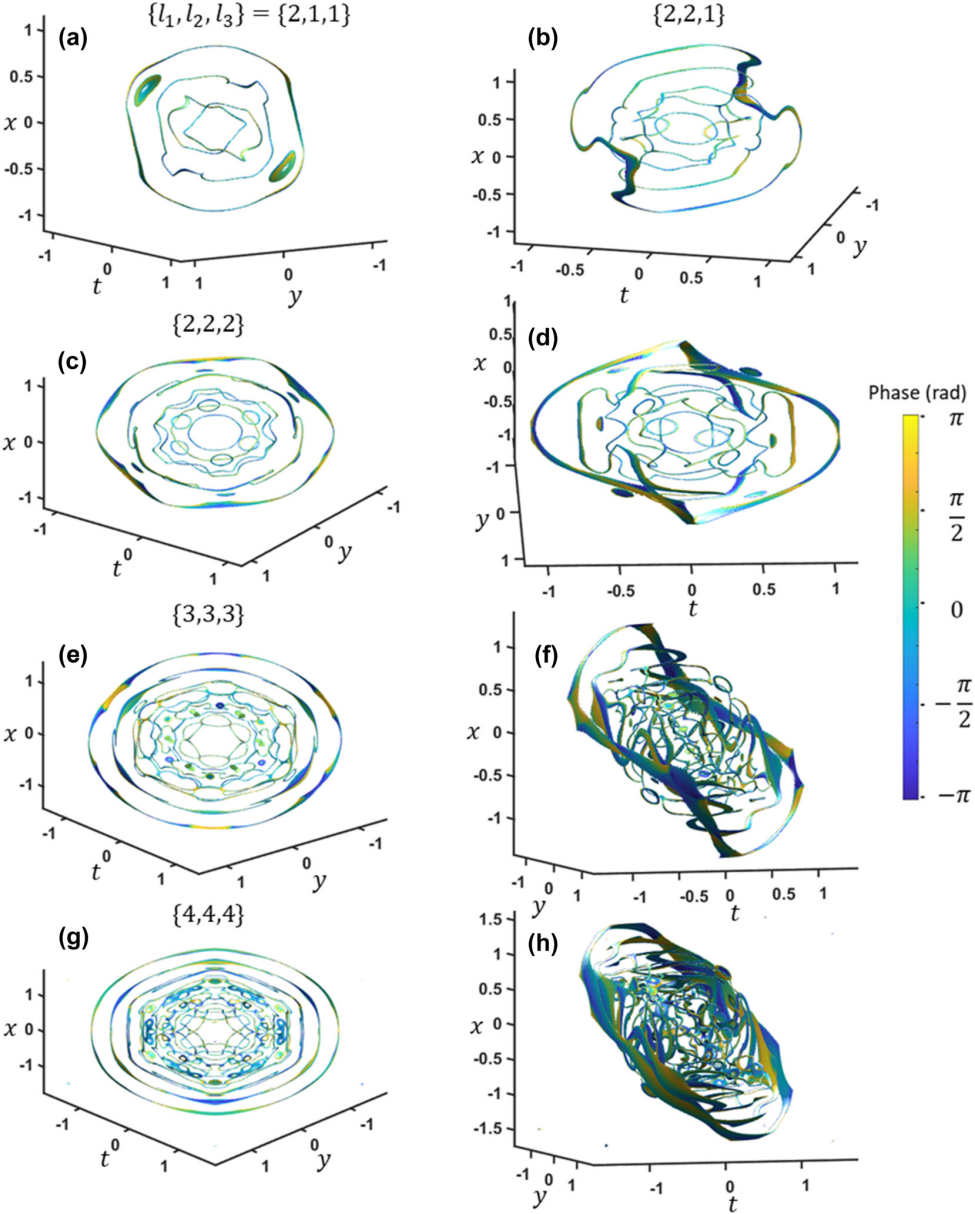
The corresponding vortex arrays at the focus for three orthogonal paraboloid profiles in the Fourier domain with paraboloid powers *l*_1_, *l*_2_, and *l*_3_, shown from a front perspective and side perspective. *x* and *y* units are in millimeters while *t* is in picoseconds.

Finally, we examine the impact of the paraboloid powers at the focus of the spatiotemporal focusing. With $l=\left\{2,1,1\right\}$ , an additional outer loop appears while the shape and orientation of all the loops shift (Figure 8a). Increasing the powers to *l* = {2, 2, 1} results in an additional loop vortex (Figure 8b). Up to this point, it appears that the total number of loops is proportional to the sum of the powers *N* = *l*_1_ + *l*_2_ + *l*_3_. However, increasing the paraboloid powers to *l* = {2, 2, 2} drastically changes the number and arrangement of loops, with six small loops emerging around the center (Figure 8c and d). As all the powers are increased together to *l* = {3, 3, 3} (Figure 8e and f) and *l* = {6, 6, 6} (Figure 8g and h), circularly symmetric kaleidoscope-like patterns emerge. Again, we find it intriguing to observe these intricate arrangements of vortices emerge by simple linear propagation.

Although generating such paraboloid null patterns experimentally in the Fourier domain can be challenging, we believe current technology advancement provides a promising opportunity for their experimental realization. For example, a recently proposed time-reversal method for arbitrary 2D+1 amplitude and phase control may be capable of generating these paraboloid null profiles, thereby facilitating loop vortex reconnections [18].

## 3 Conclusions

In conclusion, we have shown combining perpendicularly oriented paraboloid amplitude nulls in the Fourier domain of a wavepacket leads to complicated arrangements of loop vortex reconnections in the space-time domain. Different combinations of propagation, such as spherical focusing, cylindrical focusing, or temporal focusing in dispersive media, each cause unique sequences of loop vortex reconnections. Increasing the paraboloid powers significantly alters the number of vortices and complexity of the reconnection patterns. These intriguing reconnection phenomena arise from simple paraboloid nulls in the Fourier domain, which makes this more accessible to future experiments. These loop vortex reconnections occurring in linear propagation could allow deeper insight into reconnections in more complex media.

## Supplementary Material

Supplementary Material Details

Supplementary Material Details

Supplementary Material Details

Supplementary Material Details

Supplementary Material Details

Supplementary Material Details

Supplementary Material Details

Supplementary Material Details

Supplementary Material Details

Supplementary Material Details

Supplementary Material Details

Supplementary Material Details

Supplementary Material Details
