# Supplementary figures and images for: Spatiotemporal optical vortex reconnections of loop vortices
Source: Nanophotonics. 2025 Feb 4;14(6):729–39. doi: 10.1515/nanoph-2024-0594 (PMC11964299; doi:10.1515/nanoph-2024-0594)

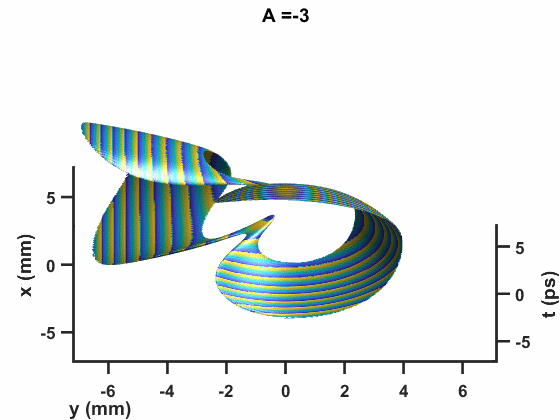

Supplement: Supplementary file 1 — Supplementary Material Details [file j_nanoph-2024-0594_suppl_001.gif]

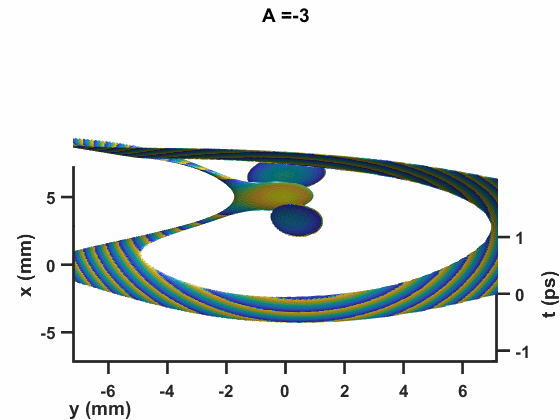

Supplement: Supplementary file 2 — Supplementary Material Details [file j_nanoph-2024-0594_suppl_002.gif]

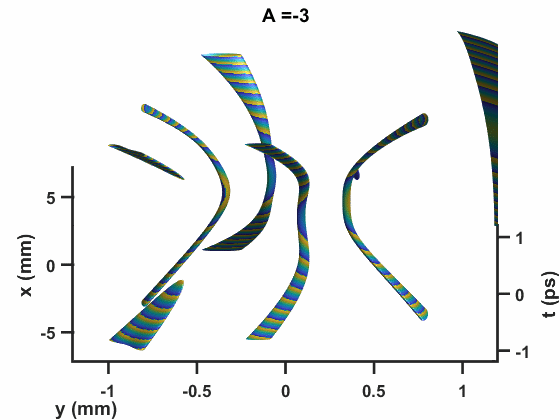

Supplement: Supplementary file 3 — Supplementary Material Details [file j_nanoph-2024-0594_suppl_003.gif]

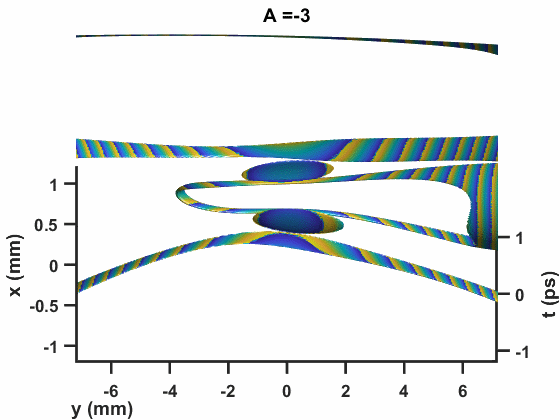

Supplement: Supplementary file 4 — Supplementary Material Details [file j_nanoph-2024-0594_suppl_004.gif]

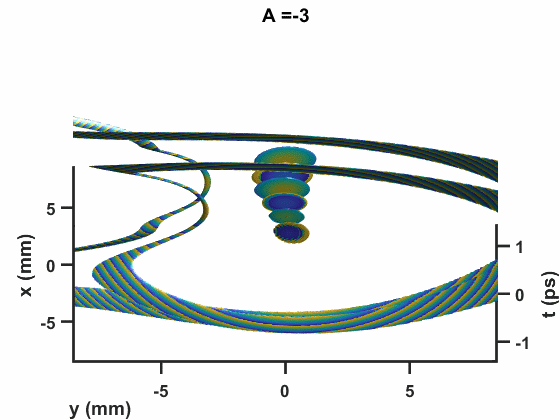

Supplement: Supplementary file 5 — Supplementary Material Details [file j_nanoph-2024-0594_suppl_005.gif]

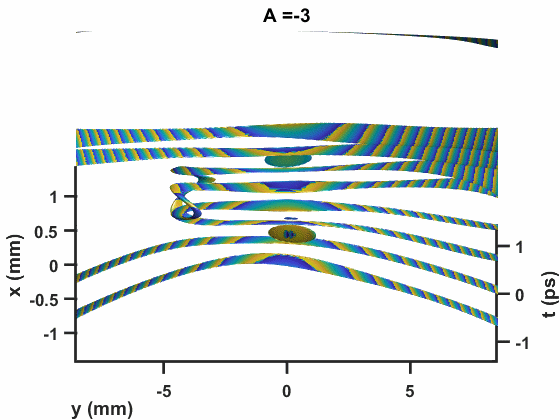

Supplement: Supplementary file 6 — Supplementary Material Details [file j_nanoph-2024-0594_suppl_006.gif]

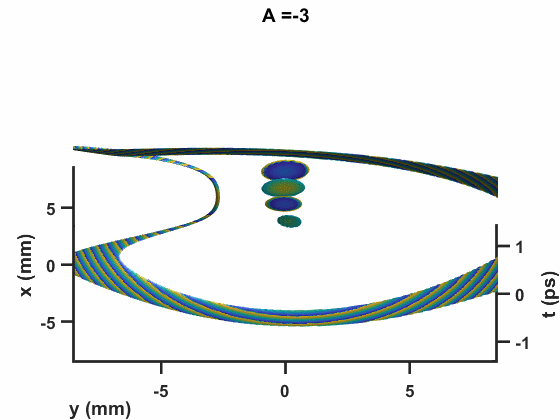

Supplement: Supplementary file 7 — Supplementary Material Details [file j_nanoph-2024-0594_suppl_007.gif]

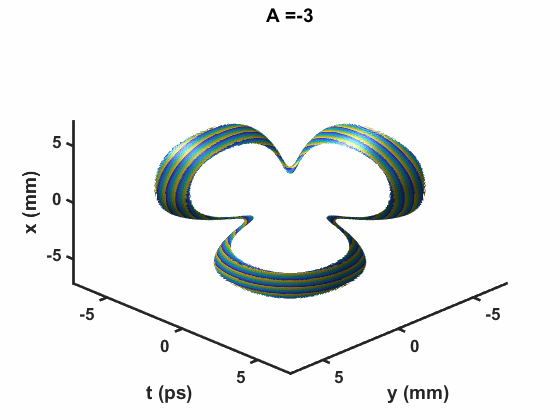

Supplement: Supplementary file 8 — Supplementary Material Details [file j_nanoph-2024-0594_suppl_008.gif]

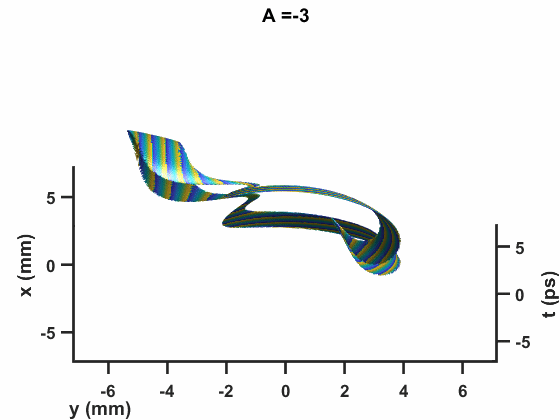

Supplement: Supplementary file 9 — Supplementary Material Details [file j_nanoph-2024-0594_suppl_009.gif]

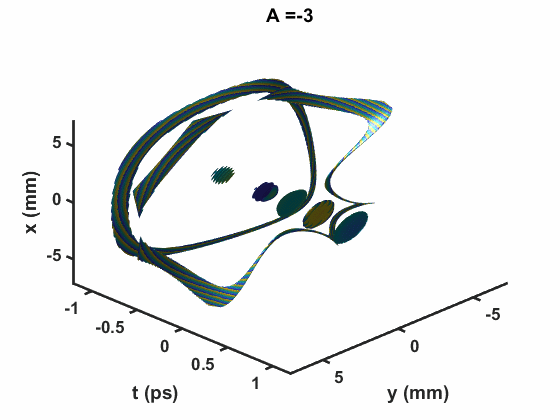

Supplement: Supplementary file 10 — Supplementary Material Details [file j_nanoph-2024-0594_suppl_010.gif]

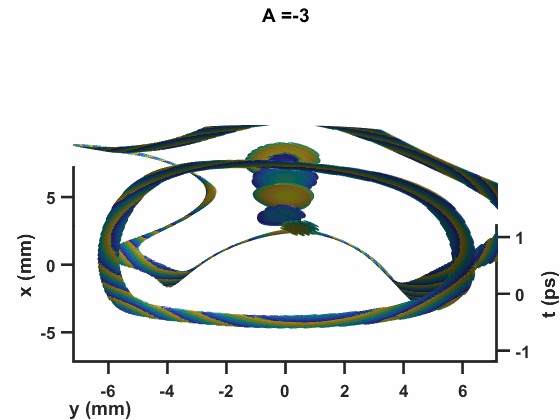

Supplement: Supplementary file 11 — Supplementary Material Details [file j_nanoph-2024-0594_suppl_011.gif]

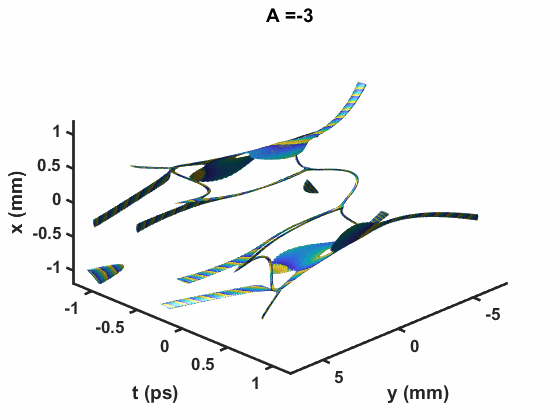

Supplement: Supplementary file 12 — Supplementary Material Details [file j_nanoph-2024-0594_suppl_012.gif]

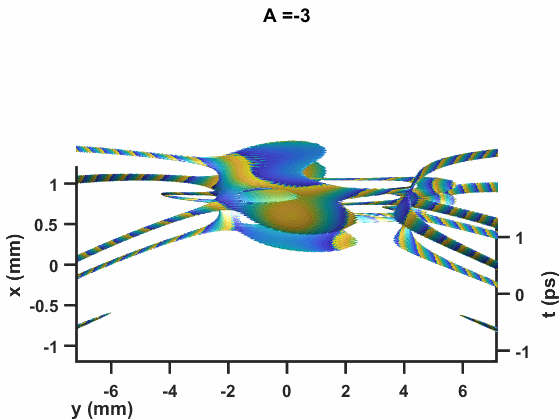

Supplement: Supplementary file 13 — Supplementary Material Details [file j_nanoph-2024-0594_suppl_013.gif]
